# Experiments on the Proliferation and Development of Leucocytes

**Published:** 1890-03

**Authors:** 

**Affiliations:** Assistant to Prof. Flemming, Kiel, Germany


### EXPERIMENTS ON THE PROLIFERATION AND DEVELOPMENT OF LEUCOCYTES.

Conducted by Dr. REINKE, assistant to Prof. Flemming, Kiel, Germany. Abstracts from an inaugural dissertation reported by Dr. Herm. Hadenfeldt, Kiel.

REINKE, assistant to Prof. Flemming, has recently made some interesting observations upon the proliferation and development of the white blood corpuscles in inflammatory conditions. His experiments lead him to the following conclusions: In the first forty-eight hours of the inflammation, mitosis does not take place in the white blood corpuscles. Difficulties attending the examination of the tissues make it impossible to distinguish positively between leucocytes and newly-formed granulation cells, since in the latter mitosis also takes place.

Reinke employed in his experiments small pieces of sponges saturated with gum arabic, and subjected to a high temperature, or better yet, the pith of sunflower stalks, which were made impervious by boiling in a six per cent. saline solution; these then, following all aseptic cautions, were introduced under the skin of animals. In these masses, which act as foreign bodies and excite an inflammation, the leucocytes enter and can be easily examined, after staining with Flemming's solution of safranin. Reinke found, in spite of many preparations which had been exposed three, six, twelve, and twenty-four hours, no mitosis occurring in the many leucocytes.

The assertion, Marchand's, that he had been able to find mitosis in one preparation, Reinke tries to disprove, in that he has also seen bodies resembling mitosis. They are, however, nothing more than leucocytes which have absorbed microörganisms. These resemble mitosis, but are certainly due to germs, since under aseptic conditions they are not to be found.

To explain the fate of the wandering cells, or leucocytes, in inflammatory tissues, Reinke employed the same methods as before. After twenty-four hours, it was observed that granulation cells were shooting into these foreign bodies, closely resembling leucocytes, and which, in later stages, developed into epitheloid tissue. Now, in order to distinguish the leucocytes from the granulation cells, and to study more closely the former, the sponges were removed after twenty-four hours. Inoculations were then made from one animal to another, until it was certain that only leucocytes were present in the preparations.

Reinke found that, in inoculations up to the sixth day, the character of the cells did not change, while in those cases where the sponges were removed after forty-eight hours after the formation of granulation cells, the appearance was an entirely different one—resembling more or less epitheloid cells.

From these experiments, he infers that the white blood corpuscles either degenerate or are absorbed secondly, that after inflamed tissues have begun to proliferate, wandering cells arise, capable of further development.

These simple and interesting experiments give positive support to the position taken long ago by Virchow, and later by Grawitz. Both authors assert that the so-called pus-cells in inflamed tissues are partly white blood corpuscles, and partly granulation-cells. Both are at first indistinguishable, but later on the former degenerate, while the latter serve to regenerate the tissue through the formation of epitheloid cells.

